# Costs of Promoting Exclusive Breastfeeding at Community Level in Three Sites in South Africa

**DOI:** 10.1371/journal.pone.0079784

**Published:** 2014-01-10

**Authors:** Lungiswa Leonora Nkonki, Emmanuelle Daviaud, Debra Jackson, Lumbwe Chola, Tanya Doherty, Mickey Chopra, Bjarne Robberstad, for the Promise-EBF Study Group

**Affiliations:** 1 Health Systems Research Unit, Medical Research Council, Tygerberg, South Africa; 2 Centre for International Health, University of Bergen, Bergen, Norway; 3 Division of Community Health, Faculty of Health Sciences, Stellenbosch University, South Africa; 4 School of Public Health, University of the Western Cape, Bellville, South Africa; 5 UNICEF, New York, New York, United States of America; University of Southampton, United Kingdom

## Abstract

**Background:**

Community-based peer support has been shown to be effective in improving exclusive breastfeeding rates in a variety of settings.

**Methods:**

We conducted a cost analysis of a community cluster randomised-controlled trial (Promise-EBF), aimed at promoting exclusive infant feeding in three sites in South Africa. The costs were considered from the perspective of health service providers. Peer supporters in this trial visited women to support exclusive infant feeding, once antenatally and four times postpartum.

**Results:**

The total economic cost of the Promise-EBF intervention was US$393 656, with average costs per woman and per visit of US$228 and US$52, respectively. The average costs per woman and visit in an operational ‘non research’ scenario were US$137 and US$32 per woman and visit, respectively. Investing in the promotion of exclusive infant feeding requires substantial financial commitment from policy makers. Extending the tasks of multi-skilled community health workers (CHWs) to include promoting exclusive infant feeding is a potential option for reducing these costs. In order to avoid efficiency losses, we recommend that the time requirements for delivering the promotion of exclusive infant feeding are considered when integrating it within the existing activities of CHWs.

**Discussion:**

This paper focuses on interventions for exclusive infant feeding, but its findings more generally illustrate the importance of documenting and quantifying factors that affect the feasibility and sustainability of community-based interventions, which are receiving increased focus in low income settings.

## Introduction

Suboptimal breastfeeding has been estimated to be responsible for 1.4 million child deaths worldwide, which represents 12% of deaths in children under 5 years of age and 44 million disability adjusted life years (DALYs) [1]. Appropriate breastfeeding can reduce the prevalence of the main causes of infant death, including diarrhoea, pneumonia and neonatal sepsis [2]. A key element of feeding guidelines is that infants should be exclusively breastfed until they are 6 months of age [3].

Evidence from a systematic review suggests that Community health workers (CHW) can be effective in improving exclusive breastfeeding (EBF) rates. This international experience is confirmed in the South African context. However, the extent to which CHWs can improve EBF rates is varied. An intervention cohort study from Kwa-Zulu Natal (VTS) reported EBF rates of 76.5% and 66.7% at 5 months for HIV negative and positive women, respectively following intensive home visit support [4]. PROMISE-EBF a cluster randomised trial implemented in three sites (Kwa-Zulu Natal, Western Cape, and Eastern Cape) was also successful in increasing exclusive breastfeeding. However, PROMISE-EBF achieved a lower level of effectiveness than the VTS study. At 12 weeks of age, the EBF prevalence in the intervention and control arms were 10.5% and 6.2% in South Africa, with a prevalence ratio (PR) of 1.72 (95% CI 1.12–2.63) [5]. In PROMISE-EBF women received 5 visits, whereas, in the VTS study the high impact of peer support was achieved with an intensive intervention with as many as 18 visits during the antenatal period until the infant was 6 months old [6].

Community-based interventions and task shifting are now high on the Millennium Development Goals (MDG) policy agenda. Large investments are being made in CHW programmes through disease specific channels; this is evidenced by the use of CHWs in HIV, TB, child health and malaria programmes [7]. Lack of skilled health workers and recent effectiveness evidence has boosted the interest in the use of lay health workers, and many countries are again investing in national programmes [8]. The increased interest is driven by an expectation that the inclusion of lay health workers will render health systems cost-effective by reaching large numbers of previously under-served people with high-impact basic services at low costs [9]. Little work has been done to estimate the cost of delivering such interventions [10].

Economic evaluations from South Africa and Uganda have shown that stand alone individual peer support is not inexpensive [6], [11]. The economic evaluations from Uganda and South Africa were conducted alongside a cluster randomised trial and prospective cohort study respectively and all costs were adjusted to 2007 prices. In the Ugandan study the cost per mother counselled was US$139 and the cost per visit was US$26 [11]. The South African economic evaluation was a cost effectiveness analysis with three scenarios. First, a full scenario which was the intervention as it was implemented under research conditions. The simplified and basic scenarios had fewer visits, 6.2 and 3 visits respectively. The simplified scenario had more clinic than home visits, and the basic scenario had no home visits and was entirely clinic based. The total costs for the three scenarios were US$14 million (full scenario), US$7 million (simplified scenario) and US$2 million (basic scenario) per year in the Kwa-Zulu Natal province [6]. The costs per month of exclusive breastfeeding for the full, simplified and basic scenarios were US$48, US$29 and US$88 respectively. The study showed that home visits have a role in EBF promotion, and the authors recommended the simplified package.

In this paper we analyse the costs of providing peer counselling through five visits at home to promote EBF up to 3 months after delivery in three South African communities. We also assessed the potential affordability of the intervention in an operational setting. This study provides evidence from an upper middle-income country in sub-Saharan Africa on costs of promoting EBF through a low intensity intervention (five visits), in a high antenatal HIV prevalence setting, where the national exclusive breast feeding prevalence is low.

## Methods

### Ethics statement

Written informed consent was obtained, and all participants were informed that they could refuse participation or withdraw from the discussions at any time. Ethical approval was granted by the University of the Western Cape.

### The trial

A cluster randomised controlled trial (RCT), known as PROMISE-EBF [Promoting infant health and nutrition in sub-Saharan Africa: Safety and efficacy of exclusive breastfeeding promotion in the era of HIV (http://clinicaltrials.gov/ct2/show/NCT00397150)], was designed to improve the rates of exclusive infant feeding (i.e. exclusive breastfeeding (EBF) at 12 weeks through the assistance of community peer supporters. PROMISE-EBF [5] was conducted in four sub-Saharan African countries, namely Burkina Faso, South Africa, Uganda and Zambia. The costing analysis for Uganda has been published elsewhere [11].

### Study area

In South Africa, the intervention was implemented in three study sites amongst HIV-positive and HIV-negative women. These sites represented a variety of settings that exist in South Africa in terms of area of residence, antenatal HIV prevalence and health systems functioning [12]. The site were a peri-urban farm area (Paarl) in the Western Cape Province, a rural area (Rietvlei) currently in Kwa-Zulu Natal but was part of the Eastern Cape at the time of the study and an urban township (Umlazi) in Kwa-Zulu Natal province. The antenatal HIV prevalence in these areas was 12.6%, 26.0% and 37.4% respectively [13].

### The intervention

During the course of routine antenatal care and hospital deliveries (96% of pregnant women), expectant mothers are ideally offered voluntary counselling and testing for HIV, and are also counselled on infant feeding choices. Optimally, at the end of routine antenatal care, HIV positive mothers were to be in a position to choose between EBF and EFF based on WHO AFASS recommendations. In the intervention clusters, the peer supporters' tasks were to recruit pregnant women, establish the mother's feeding choice and thereafter support the mother in carrying out her choice of EBF or EFF. Mixed feeding was discouraged. The intervention included at least one antenatal home visits by peer supporter, plus four visits after delivery at 1, 4, 7 and 10 weeks, with the possibility of an extra visit when necessary. In the control clusters, mothers also received visits; however, the content of their visits was not on feeding. It was on accessing social grants.

A total of 18 female peer supporters were employed in the infant feeding support intervention arm: five in Paarl, six in Rietvlei and seven in Umlazi. Each peer supporter had a designated geographic area. Peer supporters had completed at least 12 years of schooling, had an interest in child health, prior experience of community involvement and lived within the selected trial clusters. There was neither an age limit nor a requirement for them to have personally breast fed. Peer supporters had to successfully complete a literacy and basic counselling skills assessment. They received five training sessions. The first session was a five-day WHO/United Nations Children's Fund (UNICEF) HIV and Infant Feeding Counselling Course [3]. The subsequent four training sessions were developed in response to needs identified by peer supporters as the intervention progressed. These one- or two-day sessions covered topics about HIV (disclosure and transmission), computer training and care giving.

The cluster size was determined by the estimated local fertility rates and appropriate number of women needed for the trial. The research team identified rational distinct geographic units containing 3000 women of childbearing age in both Paarl and Umlazi, and 1500 women in Rietvlei. Peer supporters commenced work in September 2005 and completed follow-up of all women recruited for peer support in December 2007.

### Peer support supervision

One supervisor per site was appointed to manage and support 10–14 peer supporters. The supervisors had varied skills, ages and backgrounds. They were all experienced field researchers. Their role was to help the peer supporters, and to encourage them to give high quality and consistent counselling. Peer supporters had monthly group meetings with a supervisor, at least one individual contact session each week (telephonically or face-to-face) and were observed counselling a mother during a home visit at least once a month. In addition, supervisors visited a random sample of mothers to verify that peer support had taken place according to schedule. The supervisor workload varied across the three sites.

Supervisors were themselves supervised telephonically or in person by a qualified social worker from the research team, who liaised directly with senior research staff. This person visited the site once a month and served as an intermediary between peer supporter supervisors and the research project manager. The senior research staff had training in nursing and maternal and child health.

Peer supporter supervisors received six training courses. They received two sessions of trainers courses: a five-day WHO/United Nations Children's Fund (UNICEF) HIV and Infant Feeding Counselling Course, [3] or a course on accessing child grants and other social services. The supervisors' subsequent training was similar to that of peer supporters. However, the supervisors did not receive computer training or workshops on care giving. Instead, they received training on supervision and study operating procedures.

### The economic evaluation

Full economic evaluation is defined as a comparison of two or more interventions in terms of their costs and consequences [14]. Thus, the comparison was between the promotion of exclusive breastfeeding using home-based individual peer support and the status quo and the costs of the status quo were assumed to be zero. The costing analysis was conducted alongside the RCT, which was implemented by the University of the Western Cape (UWC), South African Medical Research Council (SAMRC) and Health Systems Trust (HST).

### Costing

The costing was conducted from a provider's perspective. Promise-EBF costs were measured prospectively throughout the duration of the trial. In addition, we reviewed the projects financial records – budget and expenditure reports from all three (UWC, SAMRC, and HST) institutions. We estimated economic costs approximating the actual opportunity value of the resources [14]. For goods and services paid for by the Promise-EBF trial, valuation was done using the actual market prices. For non-market goods, including donated staff time, office space, furniture and vehicles that were inherited from other projects, prices were estimated by establishing the replacement costs of each good in its current condition and working out the remaining useful life years. In the case of donated staff time we used an equivalent wage rate for their level of skill in the market. Since the perspective of the study is the local health care provider, rather than society, adjustments for taxes (e.g. Value added tax) and subsidies were not required.

Promise-EBF was a research project, and therefore some resources were used for both research activities and the intervention. Our primary concern was to estimate the costs of establishing and running a peer-counselling intervention to promote exclusive infant feeding, and not to estimate research costs. Therefore, we allocated joint costs (research and intervention) by interviewing project managers about the proportion of time that the cost item was used for during the intervention, and based on this, excluded the research-related costs. Each salary included reflecting the proportion of time spent on the intervention (50% of the site supervisor and 25% of the project manager's time). The use of drivers varied between sites. Umlazi had two drivers, each of whom spent 30% of their time on the intervention. In Rietvlei, the driver spent 30% of his time on the intervention, while in Paarl the driver spent 85% of his time. Vehicles, office equipment and supplies were included under supervision since they were used for the supervision of peer supporters.

The study utilised a combination of activity-based costing and an ingredients approach [15], [16], and the costing exercise was based on four main categories of activities: set-up, training, peer support and peer support supervision (Table 1). For each activity, all inputs necessary for producing that activity were identified, measured and valued.

**Table 1:** Description of project activities with respect to types of input requirements.

Activity	Description of inputs
***1. Start up***	
Once –off	Manual development and intervention design
Repeatable	Peer supporter materials, recruitment of peer supporters, training of peer supporter supervisor and peer supporters
***2. Training (excludes start-up training)***	Training of replacements, HIV (disclosure and transmission) and workshops on care giving and discipline
***3. Peer support***	Peer supporter salaries, mobile phone vouchers, transport (re-imbursements) and household incentives
***4. Peer support supervision***	Peer supporter supervisor salaries, site supervisor, project manager, drivers salaries, vehicles, office equipment and office supplies

Within the set-up costs, some costs were categorised as once-off (i.e. costs incurred only once for the program) and repeatable costs (i.e. to be repeated if the program is rolled out to other districts). Set-up costs are expected to yield benefits for several years after project initiation, and were therefore annuitised [17], [18]. Once-off set-up costs included costs related to planning and designing the intervention, and the local adaptation of the WHO/Infant feeding manual [3]. Repeatable costs were recruitment of peer supporters and the initial training of peer supporters and supervisors. The once-off costs were annuitised over 10 years to reflect their potential for use, not only in scale-up but also in other settings. The repeatable costs were annuitised over 5 years. Refresher training and training of new recruits were included in recurrent costs.

Capital costs were calculated using the replacement value of each item, the estimated number of useful life years and were annuitised using a discount rate of 8% (the interest rate of South African long-term government bonds), which is consistent with other studies in South Africa [19]. We assumed useful life years of 7 years for vehicles, and 5 years for other capital items such as computers, printers, copiers and furniture [20], [21]. Capital equipment with a unit price of less than US$100 were treated as recurrent costs [16].

The purchasing power of money diminishes with inflation, and costs were adjusted for time using the consumer price index, excluding mortgage bonds [22] with 2007 as the base year. Rands were converted to US dollars (US$) using the average exchange rate for 2007 (R7.9 to US$1) [23].

Data sources included time-use logs filled by peer supporters and data from the accounting systems of the three institutions that jointly implemented the intervention. The project manager, supervisor and peer supporter supervisors were interviewed to validate the expenditure information.

The costs of implementing peer support included four main categories. First was the monthly peer supporter stipend of US$127, which increased to US$152 in the second year of the trial. Secondly, each peer supporter had a mobile phone and was provided with airtime vouchers worth US$14 per month to communicate with the mothers. Thirdly, peer supporters in two sites (Rietvlei and Umlazi) used public transport to reach some of their clients due to long travel distances and were reimbursed for these costs. The final cost was for a once-off household incentive of one food parcel worth US$5 per household receiving peer support.

The costs of peer supporter supervision included the salaries of supervisors, project managers and drivers.

The post set-up training included training of new peer supporters due to staff turnover and continuing education for existing peer supporters. Overhead costs included all the inputs not directly involved in delivering the intervention (Table 1).The total cost of the peer support intervention was the sum of all the activities' costs. The average costs per woman and per visit were also calculated.

### Adaptations made for an operational scenario

PROMISE EBF was a vertical stand-alone project. We present cost estimates that are as representative as possible for a real operational set-up of a peer support intervention. We do this by adjusting the PROMISE EBF estimates of resource use and values for start-up activities, peer supporter supervision and the actual peer supervision (Table 2).

**Table 2:** Description of inputs varied in the Promise-EBF scenario and the alternative operational set up with integration into existing community health worker programmes.

Promise-EBF	Operational Scenario
**Personnel**	**Personnel**
Project Manager	District deputy director (no incremental costs assumed)
Site supervisor	Site supervisor
Peer supporter supervisors	Professional nurse
Peer supporters	Peer supporters
Drivers	
**Start-up costs**	**Start-up costs**
Once-off (annuitised using 3 years)	
Repeatable (annuitised using 3 years)	Repeatable (annuitised using 5 years)
**Peer support supervision**	**Peer support supervision**
Six vehicles	Three vehicles (one per site)
Air travel	Air travel (excluded)
**Peer support**	**Peer support**
Household incentive food parcel	Household incentive (excluded)
**Training**	**Training**
Computer training	Computer training (excluded)

### Adjustment of inputs in start-up activities

Our focus was at the district level. Therefore, we excluded the costs of developing the intervention and a manual. These activities typically take place at national level, and thus were a one-off central activity, which will not be repeated when the intervention is rolled out.

### Adjustment of inputs in peer support supervision

In an operational South African setting, the project manager would have lower levels of skills than in Promise-EBF and would typically be an assistant director at a district level. Each district already has an assistant director and implementing this project will therefore not necessitate employing new management. Instead we assume that 25% of existing assistant directors time will be spent on the intervention, and included the corresponding proportion of an assistant director salary package.

Peer supporter supervisors salaries in the trial ranged from US$16 269 to US$29 457 per year across the three sites for being employed on a part time (50%) basis. It is difficult to estimate peer supporter supervisor salary scales in an operational scenario because in South Africa, CHWs exist in various forms with various types of supervision [7], [24]. In 2007, salaries for CHW supervisors ranged from US$5 759 to US$20 433 per year and employment contracts vary between full time and half time [25]. In the operational scenario, we reduced the peer supporter supervisor salaries to the minimum level (US$5 759), which is offered by NGOs. This choice is consistent with the recommended remuneration for supervisors in the Expanded Public Works Programme [26].

Travel costs were adjusted downward because of the fact that the study offices in all sites were far away from the communities. In a district setting, the offices will typically be much closer, and drivers' salaries were therefore excluded as they are not generally part of a district set up.

### Adjustment of inputs for peer support

In the second year of the Promise-EBF study, it was decided to provide households with a once-off incentive for receiving peer support in the form of a food parcel worth US$5. This was induced by trial conditions and would not be repeated in the operational setup. These costs were excluded, although we acknowledge that these incentives could have had an impact on the project uptake.

### Output and average costs

The impact of the intervention was measured using the number of women who received peer support and who practiced exclusive breastfeeding up to three months after delivery. The two main outputs were total number of mothers' counselled and total number of counselling sessions or visits. These were combined with total costs to calculate average costs per visit and per mother counselled. We used these to approximate the cost per month of exclusive breast feeding (MEBF) which was defined as our primary health outcome of interest. MEBF were the sum of the duration, in months, that a child was exclusively breastfed. The cost per MEBF was expressed as the total cost divided by total MEBF at three months.

## Results

### Outputs and average costs

During the project period of 2.5 years, a total of 1725 women were followed up, with 526, 611 and 588 from Paarl, Rietvlei and Umlazi, respectively. On average, women received four visits in Paarl and Rietvlei, which was one visit below the target. In Umlazi, women on average received the planned five visits.

### Promise-EBF scenario

The total economic costs in the Promise-EBF scenario were US$393 656. The average costs per woman and per visit in the Promise-EBF scenario were US$228 and US$52 respectively (Table 3). The MEBF were estimated to be 169 at 12 weeks, based on 24 hour recall of feeding practice. The costs per MEBF were US$ 2 250. Peer support supervision was the main cost driver and accounted for 55% of total costs (Table 3). Personnel costs accounted for 80% of the supervision costs; followed by office supplies and vehicle maintenance which accounted for 9% and 7% respectively. Communication and equipment accounted for the remaining 5%.

**Table 3:** Economic costs per site of the total cost of the intervention, cost per visit and cost per woman.

	Paarl				Rietvlei				Umlazi				Combined			
	Promise EBF		Operational		Promise EBF		Operational		Promise EBF		Operational		Promise EBF		Operational	
	$	%		%	$	%		%	$	%		%		%		%
**A. Set-up**																
One off	756				756				756				2,269			
Repeatable	1,727		1,460		1,790		1,585		1,457		1,321		4,974		4,366	
***Sub total***	***2,483***	***2***	***1,460***	***2***	***2,547***	***2***	***1,585***	***2***	***2,213***	***1***	***1,321***	***1***	***7,243***	***2***	***4,366***	***2***
**Average cost/woman (A)**	**5**		**3**		**5**		**3**		**4**		**3**		**14**		**8**	
**Average cost/visit (A)**	**1**		**1**		**1**		**1**		**1**		**1**		**3**		**2**	
**B. Implementation costs**																
**Overheads**																
Office rentals	6,814				4,210				17,781				28,805			
vehicle insurance	989				1,714				935				3,638			
Other	1,500				2,926				3,217				7,643			
***Sub total***	***9,303***	***9***	***6,329***	***10***	***8,850***	***7***	***8,354***	***10***	***21,933***	***14***	***8,987***	***10***	***40,085***	***10***	***23,671***	***10***
**Training**																
Ongoing	2,782		1,416		6,935		4,228		6,056		2,908		15,774		8,552	
Replacement	4,303		1,654		1,073		1,073		1,971		1,443		7,347		4,170	
***Sub total***	***7,085***	***6***	***3,070***	***5***	***8,008***	***6***	***5,301***	***7***	***8,027***	***5***	***4,352***	***5***	***23,121***	***6***	***12,723***	***5***
**Peer support**																
Personnel	21,635		21,635		25,962		25,962		31,283		31,283		78,881		78,881	
Communication	2,120		2,225		2,544		2,682		3,078		3,624		7,743		8,532	
Travel	-		-		1,495		1,495		1,262		1,262		2,757		2,757	
Materials	293		188		151		13		1,763		1,217		2,207		1,418	
Household incentive	4,133		-		4,632		-		4,801		-		13,566			
***Subtotal***	***28,181***	***26***	***24,049***	***37***	***34,785***	***28***	***30,153***	***36***	***42,187***	***26***	***37,386***	***43***	***105,153***	***27***	***91,588***	***39***
**Peer support supervision**																
Personnel	52,201		22,003		54,950		22,003		68,122		22,003		175,274		66,008	
Vehicle maintenance	2,400		2,400		8,809		8,809		3,201		3,201		14,410		14,410	
Communication	1,059		660		1,059		660		1,276		745		3,393		2,066	
Office supplies	4,529		4,529		3,954		4,277		9,996		9,996		18,480		18,802	
Capital	1,795		949		2,256		1,458		2,444		874		6,495		3,281	
***Sub-total***	***61,985***	***57***	***30,542***	***47***	***71,029***	***57***	***37,207***	***45***	***85,039***	***53***	***36,819***	***41***	***218,053***	***55***	***104,567***	***44***
***Total (B)***	***106,555***	***98***	***63,990***	***99***	***122,671***	***98***	***81,015***	***98***	***157,187***	***99***	***87,544***	***99***	***386,413***	***98***	***232,548***	***98***
***Average cost/woman (B)***	***203***		***122***		***234***		***154***		***299***		***167***		***736***		***443***	
***Average cost/visit (B)***	***47***		***28***		***54***		***36***		***69***		***38***		***170***		***102***	
**Total (A+B)**	**109,038**	**100**	**65,450**	**100**	**125,218**	**100**	**82,600**	**100**	**159,400**	**100**	**88,865**	**100**	**393,656**	**100**	**236,914**	**100**
**Average cost/woman (A+B)**	**208**		**125**		**205**		**135**		**272**		**151**		**228**		**137**	
**Average cost/visit (A+B)**	**48**		**29**		**52**		**34**		**58**		**32**		**52**		**32**	

The peer support activity was the second largest cost driver, representing 27% of the costs. Personnel costs accounted for 75% of peer support costs. In the second year of the study, the food parcels, valued at US$5 each accounted for 13% of the peer support costs. Communication in the form of mobile phone airtime amounted to 7% of peer support costs. Peer supporters in the Rietvlei and Umlazi sites resorted to public transport when the distances between households were not within walking distance. Although peer supporters had to reside in the same clusters as the pregnant women in order to avoid transport expenditure, distances between households proved to be more than envisaged, especially in the rural site. In Paarl, peer supporters were able to conduct all the visits on foot. Travel costs for peer supporters accounted for 3% of the total costs.

Training accounted for only 6% of the total costs. In total, 25 training sessions were conducted. Of these, four were initial training courses during the set-up phase, 16 were refresher courses and five were for staff replacement.

Overhead costs accounted for 11% of the total costs, of which office rental was the highest cost driver with a share of 72%.

We report the attrition of peer supporters in both arms in order to capture the potential magnitude of this problem. This trial employed 38 peer supporters (counting both the control and intervention arms), of which, one-third resigned during the study period. Attrition varied by site: 60% in Paarl, 36% in Rietvlei and 14% in Umlazi. In Paarl, 6 of the 10 peer supporters resigned and had to be replaced, and in Rietvlei, 5 out of 14 resigned. In Umlazi, none of the peer supporters resigned, but four were lost due to illness. It was only in Umlazi that back-up peer supporters were trained at the start of the study, meaning that peer supporters could be replaced immediately. In both arms, peer supporters were required to conduct the same number of visits, their stipends were of the same value and they were supervised by the same person. Peer supporters left for better paying employment opportunities.

### Comparison of costs between sites

The cost pattern in the individual sites was similar to that of the combined analysis, although the percentage share of costs for each activity varied somewhat (Table 3). Peer support supervision was the cost driver in all three sites. Overhead costs were lowest in Rietvlei and the highest in Umlazi. Paarl had the least peer support costs, while Umlazi had the least peer support supervision costs.

Table 4 presents the estimated workload of peer supporters per month using both quantitative and qualitative information on time use. Each peer supporter was expected to recruit 7 new mothers per month. It was estimated that recruiting 7 mothers would translate to peer supporters following up 21 mothers per month (expected follow-up time was 3 months). A peer supporter would be seeing mothers at different stages of pregnancy/postnatal period at any one time. The average total number of hours spent on peer support per month was 50, 75 and 59 for Paarl, Rietvlei and Umlazi respectively. The rural site had the longest travelling time with an average of 84 minutes between visits.

**Table 4:** Peer supporters' time use.

	Paarl			Rietvlei			Umlazi			Combined	
Activities of peer supporters	Intensity (activities per month)	Time per activity (min)	Total time per month (min)	Intensity (activities per month)	Time per activity (min)	Total time per month (min)	Intensity (activities per month)	Time per activity (min)	Total time per month (min)	Time per activity (min)	Total time per month (min)
Recruitment	10	60	600	10	60	600	10	60	600	60	600
Visit	21	60	1 260	21	60	1 260	21	60	1 260	60	1 260
Travel	21	15	315	21	85	1 785	21	40	840	47	980
Monthly meeting	1	240	240	1	240	240	1	240	240	240	240
Recording and planning	5	120	600	5	120	600	5	120	600	120	600
Total time (min)			3 015			4 485			3 540		3 680
Total time (hours)			50			75			59		61

### Operational scenario

In the operational scenario, the total costs were reduced by 38%, which amounted to US$ 236 914. The average costs per woman and per visit were US$137 and US$32 respectively (Table 3). In the operational scenario, peer support supervision accounted for 44% of the costs and was a less influential cost driver in both absolute and relative terms. The major change in the composition of the costs of the program that results from this reduction is in the mix of peer support and supervision costs. They go from accounting for 27% and 55% of total costs, respectively, under the Promise EBF scenario, to 39% and 44%, respectively, under the operational scenario. The biggest modifications that produce these changes for the peer support component are: the household incentive is eliminated in going from the Promise EBF to the operational scenario, which accounts for 98% of the 22% reduction in peer support costs—going from US$105,153 to US$91,588. The biggest modifications that produce these changes for the supervision component are: personnel costs are reduced from US$175,274 to $66,008.

## Discussion

The operational cost of US$137 per woman is substantial compared to an average of US$38 per uninsured person per annum in 2007 (time of the study) for public sector expenditure on non-hospital primary health care (PHC) services in South Africa [27]. Therefore, community-based peer support may not provide less expensive services compared to PHC services in this context. It should be noted that the average cost for PHC per uninsured person includes all ages, gender, and population groups. Recent mothers are a group with a high need for PHC, and are expected to consume much more PHC resources than the average client.

Several factors may have increased the costs of Promise-EBF. Firstly, Promise-EBF was a geographically limited intervention and could therefore not benefit from potential economies of scale. Secondly, peer supporters in Promise-EBF delivered a single intervention, promotion of exclusive breastfeeding. CHW programmes that are focused on a single intervention (either curative or preventive) have been found to be expensive compared to those that cover multiple interventions [28]. Implementing Promise-EBF at a larger scale and increasing the tasks of CHWs' could result in unit cost reductions. Another factor that can result in unit cost reductions is the duration of time that the programme has been in existence. Makan and Bachman [28] found that CHWs and managerial staff became more efficient over time.

The cost per woman of US$ 220 for the Promise-EBF intervention in South Africa was nearly twice the cost per woman for a comparable intervention (US$139) in Uganda [11], but slightly lower compared to Zambia (US$ 233). [Chola L et al; Unpublished] The higher cost per woman in South Africa and Zambia indicate higher cost structures in these countries. For instance, CHWs (peer supporters) in South Africa were remunerated with US$152 per month, whereas in Uganda they received a stipend of US$20 per month.

The numbers of home visits (i.e. the intensity of the intervention) have been shown to influence total costs. The simplified scenarios presented by Desmond *et al* (discussed in the introduction of this paper) clearly demonstrated that the reduction in number of visits reduces total costs [6]. The resulting dilemma for policy makers and researchers in low-and middle-income countries is to what extent one can modify a highly intensive intervention that has been shown to be effective without losing its effectiveness.

The Promise-EBF trial had a total of five visits per mother: Women who received peer support were more likely to breast feed than those who did not receive the support [5]. Even though the effect was statistically significant, the absolute increase of 4.3% is small. Qualitative research [29] on the experiences of peer supporters in this study revealed that peer supporters spent considerable amounts of time negotiating entry into households and building a trusting relationship with the mothers. In addition, they found it challenging to convince mothers to not mix-feed. The challenges experienced by peer supporters raise questions about whether five visits were sufficient in the South African context.

In Promise-EBF, peer supporters were employed with the same stipend as those working in the public sector. In contrast, in the Bland et al [4] study trained lay counsellors received a monthly payment of US$413 [Bland R, South Africa, personal communication] which is three times more than the amount paid to the peer supporters in the Promise-EBF trial. Many research programmes remunerate their employees at levels higher than in the health system, which impacts positively on motivation, retention and skill level [30]. Attrition represented a substantial practical challenge during the project period. High turnover of CHWs has been documented in other programmes and threatens the continuity of even the successful programmes [31].

The option of expanding the tasks of existing multi-purpose CHWs to include EBF promotion, as suggested by Desmond *et al*, requires careful planning in South Africa. Firstly, it will only be feasible if the existing CHWs have spare time. Breastfeeding support requires scheduled counselling visits, and adherence to these is strongly associated with adherence to EBF [32]. In Paarl, the working hours of the peer supporters were one-third of full-time hours, nearly one-half in Rietvlei and about 40% in Umlazi. It may not be appropriate to increase the workload without increasing remuneration.

The South African National Department of Health is currently piloting primary healthcare (PHC) reengineering. The reengineering of PHC in South Africa has a strong focus on community based services through PHC outreach teams. These teams include multi skilled CHWs. Thus, there is now an opportunity for EBF promotion to be part of a broader integration and coordination of CHW services. If the promotion of EBF is to be part of PHC reengineering, both CHW's and their supervisors would have to receive sufficient training on supporting pregnant women on appropriate infant feeding choice while they are pregnant and EBF support after delivery. Effective support for EBF requires multiple visits. Thus, CHW supervision should ensure that CHW's work plan includes scheduling subsequent visits.

CHWs have been shown to be effective when functioning within a limited scope [33] with specific tasks and duties [34]. A rigorous evaluation of the PHC reengineering in South Africa, will be useful in understanding the effectiveness of multi skilled CHWs in improving health outcomes.

This costing analysis was strengthened by prospective and rigorous process evaluations, which were qualitative studies. These studies captured experiences of peer supporters [29], [35] peer supporter supervisors [36] and mothers who received peer support [35]. These evaluations enriched this costing exercise and we were able to cost the intervention as it was implemented.

This costing exercise was conducted from a provider's perspective, meaning that only costs incurred by the provider were considered. This approach leaves out the value of mothers' time of receiving the intervention. Costs that largely affect recipients of interventions are travelling costs and the opportunity cost of their time (i.e. the time required to receive the intervention). Given that the intervention was delivered at home, it is unlikely that women incurred travelling costs. The intervention only required that women set aside time to receive support from a peer supporter. These visits did not exceed one hour. Therefore, women had to forgo a small portion of their time to receive the intervention. It is reasonable to assume that taking a provider's perspective did not leave out substantial costs from the women's point of view. The study also did not consider the cost-savings represented by reduced incidences of diarrhoea and other childhood diseases such as pneumonia. [37] This was outside the scope of the study, but means that the reported costs are potentially overestimated, if one considers evidence from other studies. However, in Promise-EBF there was no effect on diarrhoea [5].

Our method of separating research and routine implementation could have been strengthened through careful observation in a time-motion study.

### Conclusion

Investing in the promotion of exclusive infant feeding as a stand-alone peer support service such as the Promise-EBF requires a substantial financial commitment from government, with a cost of US$228 per pregnant woman. It is therefore important to consider possible avenues of cost reduction, such as integrating infant feeding peer support into existing CHW programmes. We demonstrate how the cost per woman can be reduced by about 40% through integration with existing CHW services. Too little is known about how well breastfeeding can be promoted in an integrated set-up, and analysis of the impact of alternative implementation schemes, combined with economic evaluation, is essential in assessing whether the added costs are reasonable compared to improvements in child health.

We believe the relevance of this paper extends beyond the particular intervention of promoting EBF, to document and quantify factors that in general affect the feasibility and sustainability of community-based interventions. This is important knowledge for sub Saharan Africa, where increasing focus is put on community-based interventions.
